# Oriental theileriosis in dairy cows causes a significant milk production loss

**DOI:** 10.1186/1756-3305-7-73

**Published:** 2014-02-19

**Authors:** Piyumali K Perera, Robin B Gasser, Simon M Firestone, Garry A Anderson, Jakob Malmo, Gerry Davis, David S Beggs, Abdul Jabbar

**Affiliations:** 1 Faculty of Veterinary Science, The University of Melbourne, Parkville, Victoria 3010, Australia; 2 Maffra Veterinary Centre, Maffra, Victoria 3860, Australia

**Keywords:** *Theileria orientalis*, Dairy cattle, Milk production, Milk fat and protein, Australia

## Abstract

**Background:**

Oriental theileriosis is a tick-borne, protozoan disease of cattle caused by members of the *Theileria orientalis*-complex. Recent outbreaks of this disease in eastern Australia have caused major concerns to the dairy and beef farming communities, but there are no published studies of the economic impact of this disease. On a farm in Victoria, Australia, we assessed whether oriental theileriosis has an impact on milk production and reproductive performance in dairy cows.

**Methods:**

Blood samples collected from all 662 cows on the farm were tested using an established molecular test. For individual cows, milk production and reproductive performance data were collected. A clinical assessment of individual cows was performed. Based on clinical findings and molecular test results, the following groups of cows were classified: group 1, with cardinal clinical signs of oriental theileriosis and molecular test-positive for *T. orientalis*; group 2, with mild or suspected signs of theileriosis and test-positive; group 3, with no clinical signs and test-positive; and group 4, with no clinical signs and test-negative. Milk production and reproductive performance data for groups 1, 2 and 3 were each compared with those for group 4 using linear and logistic regression analyses, respectively.

**Results:**

At 100 days of lactation, group 1 cows produced significantly less milk (288 l; *P* = 0.001), milk fat (16.8 kg; *P* < 0.001) and milk protein (12.6 kg; *P* < 0.001) compared with group 4. At this lactation point, group 2 also produced significantly less milk fat (13.6 kg; *P* = 0.002) and milk protein (8.6 kg; *P* = 0.005) than group 4. At 305 days of lactation, group 1 cows produced significantly less milk (624 l; *P* = 0.004), milk fat (42.9 kg; *P* < 0.001) and milk protein (26.0 kg; *P* < 0.001) compared with group 4 cows. Group 2 cows also produced significantly less milk fat (21.2 kg; *P* = 0.033) at this lactation point. No statistically significant difference in reproductive performance was found upon pairwise comparisons of groups 1–3 with group 4 cows.

**Conclusions:**

The present findings demonstrate that clinical oriental theileriosis can cause significant milk production losses in dairy cattle.

## Background

*Theileria* spp. are protozoan (apicomplexan) parasites that are transmitted by ixodid ticks and cause theileriosis in animals, including domestic and wild ruminants. Theileriosis is primarily limited to tropical and subtropical regions of the world [1]. Traditionally, theileriosis caused by one or more members of the *Theileria orientalis* complex [2-4] was believed to have a limited impact on the health of cattle in endemic regions compared with *T. parva* and *T. annulata*[3,5]. To date, eight distinct genotypes of *T. orientalis* (designated *chitose* or *type* 1, *ikeda* or *type* 2, *buffeli* or *type* 3 and *types* 4–8) have been defined using PCR-based tools [6-8]. In the Asia-Pacific region, mainly the genotypes *ikeda* and *chitose* of *T. orientalis* have been linked to clinical outbreaks of oriental theileriosis in beef and dairy cattle [9-14]. In these outbreaks, disease is usually manifested through cardinal clinical signs, such as high fever, anaemia, jaundice, lethargy, weakness, abortion and/or mortality [10-12].

Since 2006, there have been > 500 clinical outbreaks of this disease in cattle in Australia, mainly in the states of New South Wales and Victoria (G. Bailey and C. M. Bell, personal communication; June 2013). In Victoria alone, where more than 65% of the Australian dairy cattle occur (http://www.dairyaustralia.com.au), there have been ~ 150 outbreaks. Although recent studies [12,15-17] have provided first insights into the prevalence and distribution of *T. orientalis* infection, nothing is known about the economic impact associated with the clinical form of oriental theileriosis in affected regions of Australia. The present study assessed the impact of oriental theileriosis on milk production and reproductive performance of cows on a dairy farm in Victoria, Australia.

## Methods

### Study site and cattle

The present study was conducted on a closed dairy cattle farm (latitude 37° 54 ′S, longitude 146° 51′ E) in Maffra, East Gippsland, Victoria, Australia. Between 31 October 2012 and 1 December 2012, the average minimum and maximum environmental temperatures were 10.4°C and 22.4°C, respectively, and the highest rainfall was 19 mm (http://www.bom.gov.au). Included in this study were 662 cows maintained on pastures; most of them (*n* = 321) were Jersey x Friesian, and 99 Friesian, 10 Jersey and 173 were various dairy crossbreeds (Table 1). Cows were 2 to 14 years of age, with 51% being 3–5 years (Table 1); they calved from late July to early October each year. All cows were vaccinated as calves (up to one year) against clostridial disease (caused by *Clostridium perfringens* type D, *C. tetani*, *C. novyi* type B, *C. septicum* and *C. chauvoei*) and leptospirosis (caused by *Leptospira hardjo* and *L. pomona*), and were given a booster vaccination each year.

**Table 1:** Age and breed of the cows studied on a closed dairy farm in Maffra, Victoria

**Bleed**	**Date of collection**	**Number of cows bled**	**Number of cows with demographic data**	**Age (years) % (proportion)**			**Breed % (proportion)**			
				≤ **2**	**3-5**	≥ **6**	**Jersey × Friesian**	**Friesian**	**Jersey**	**Other cross-bred**
1	November 2012	662	603	16.8 (101/603)	51.2 (309/603)	32.0 (193/603)	53.2 (321/603)	16.4 (99/603)	1.7 (10/603)	28.7 (173/603)
2	March 2013	220	189	18.5 (35/189)	51.3 (97/189)	30.2 (57/189)	53.4 (101/189)	17.5 (33/189)	1.6 (3/189)	27.5 (52/189)

Cows were milked twice daily (5.30 a.m. and 3 p.m.). They grazed primarily on irrigated pastures and were also given 4 kg of feed supplement (i.e., primarily cracked wheat, corn and/or canola, together with pellets containing a mixture of minerals and monensin) per cow per day, equating to ~ 1.2 tonnes per cow per annum. A computer system (Dairy Data for Windows) was used to maintain records of milk yield, milk composition and somatic cell counts [18]. Data were obtained from herd tests performed on individual cows, every 2–3 weeks for the first 9 weeks following calving, and then bi-monthly for the remainder of the lactation); calving and mating dates for individual cows were also recorded. The results of pregnancy testing were also entered into this system, allowing a detailed evaluation of the reproductive performance of all animals in the herd.

### Clinical assessment of cows, and collection of blood samples

The clinical assessment of individual cows in the present study site was undertaken and/or supervised by a registered, specialist cattle veterinarian (J. M.), who has been practising for > 50 years. The cardinal signs recorded for a clinical diagnosis of oriental theileriosis were pale vulval mucous membrane, anorexia, lethargy and increased heart (≥ 76 beats per minute) and respiratory rates (numbers per minute) [10]. Mild or suspected theileriosis was inferred clinically based on pale vulval mucous membrane, weight loss and lethargy. During the entire study period, there was no clinical evidence of any other infectious diseases (including mastitis) other than oriental theileriosis on the farm; cattle on this farm were routinely monitored for mastitis.

Blood samples were collected from cows from the coccygeal vein (using an 18 gauge needle) into EDTA tubes by registered, practicing veterinarians (J. M. and G. D.). The packed cell volume (PCV) of individual samples was determined [17]. In November 2012, samples were collected from all 662 cows (first sampling). In March 2013, samples were collected from a subset of 220 cows (second sampling); this latter number was calculated based on the estimated prevalence of theileriosis, determined following the molecular testing of all 662 cows using a confidence of 95% and a desired absolute precision of 6.5% [19].

### Molecular testing for *T. orientalis*

Genomic DNAs were extracted from individual blood samples using the DNeasy blood and tissue kit (cat. no. 69506, Qiagen, USA) following the manufacturer’s protocol. A portion (344 bp) of the major piroplasm surface protein (*MPSP*) gene was amplified by PCR from genomic DNA using the primer pair *mpsp*-AJ-F and *mpsp*-AJ-R1 [15]. In addition, for some of the samples from second blood sampling, a portion (776 bp) of the small subunit of nuclear ribosomal RNA (*SSU rRNA*) gene was amplified by PCR from genomic DNA using the primer pair *SSU*-F and *SSU*-R [12]. For each set of PCRs, negative (no-DNA) and known positive controls (*T. orientalis*) were included. Following PCR, 5 μl of each amplicon were examined on 1.5% w/v agarose gels, which were stained with ethidium bromide and then photographed (GelDoc, BioRad). All samples that tested negative were retested after diluting genomic DNAs (1:10) in water, as described previously [17], to exclude inhibition in PCR by blood constituents. In order to display sequence variation within and among amplicons, single-strand conformation polymorphism (SSCP) analysis [20] was employed as described previously [15,17]. Aliquots of amplicons (*n* = 1–5) representing each unique SSCP profile were taken, treated with shrimp alkaline phosphatase and exonuclease I (Fermentas Inc., USA) [21] and then subjected to bi-directional, automated sequencing (BigDye® Terminator v.3.1, Applied Biosystems, USA) using (separately) the same primers employed in PCR. Amplicons representing an SSCP profile inferred to be a composite of other profiles were individually cloned and then sequenced using an established protocol [15] to determine the genotypes present. The quality of individual sequences was assessed using the program Geneious Pro 5.6.5 (Biomatters Ltd., Auckland, New Zealand), aligned using Clustal X 2.0.10 [22] and subjected to BLASTn analysis (http://blast.ncbi.nlm.nih.gov) to establish the best matches to nucleotide sequences available in the GenBank database.

### Classification of four groups of cows

Based on clinical findings and molecular test results, the following groups of cows were classified: group 1, with cardinal clinical signs of oriental theileriosis (see clinical assessment) and molecular test-positive for *T. orientalis*; group 2, with mild or suspected signs of theileriosis and test-positive for *T. orientalis*; group 3, with no clinical signs (subclinical) and test-positive for *T. orientalis*; and group 4, with no clinical signs and test-negative for *T. orientalis*.

### Collection of data on milk production and reproduction, and analyses

For individual cows, milk production was assessed. Specifically, milk volume (in l), milk fat (kg), milk protein (kg) and milk solids (kg; milk fat plus milk protein) were measured for the majority of the herd, every 2–3 weeks for the first 9 weeks following calving, bi-monthly for the remainder of the lactation, and then calculated for 100 and 305 days of lactation. Milk production data for groups 1, 2 and 3 at 100 days and 305 days of lactation were each compared with those for group 4. Linear regression analysis was conducted using statistical software IBM SPSS Statistics 22. To remove the confounding effect of age on the results, milk production data were adjusted according to age category (≤ 2 years, 3–5 years or ≥ 6 years of age). A *P*-value of <0.05 was considered as statistically significant. The losses caused by a reduction in milk production were calculated in Australian (AUD) or US (USD) dollars, according to current milk prices (i.e., 32.4 cents per litre) published in September 2012 by the Dairy Farmers Milk Cooperative, Australia.

For individual cows, reproductive performance was also monitored and evaluated. Specifically, submission rate by 21 days (SR21d), first service conception rate (CR1), in-calf by 6 weeks (ICR6w), in-calf by 14 weeks (ICR14w) and not in-calf at 21 weeks (NIC21w) were calculated. Pairwise comparisons were made between cow groups 1–3 and group 4 (= negative reference), and logistic regression analysis was conducted using IBM SPSS Statistics 22. All data were adjusted according to cow age category to remove any possible confounding effect of age on the results. Additionally, CR1 was adjusted for the calving-to-service interval (CS1) and CS1 squared (CS1sq). In addition to age categories, ICR6w, ICR14w, NIC21w and SR21d were adjusted for the calving-to-mating start date (CMSD) interval and CMSD squared (CMSDsq) [23]. A *P*-value of <0.05 was considered as statistically significant.

## Results

### Molecular identification of *T. orientalis*

Bloods from the first sampling (November 2012) were tested. Amplicons (partial *MPSP*) of the expected size (344 bp) were produced from 45.5% of the 662 blood DNA samples (Table 2). Based on SSCP analysis of all 301 amplicons, three distinct profiles were recorded; the profiles 1, 2 and 3 represented 294, 5 and 2 of the 301 samples, respectively (Table 2). Aliquots of multiple (3–5) amplicons representing each of the profiles 1–3 were sequenced. The sequences of all amplicons representing each profiles 1 and 2 represented *T. orientalis* genotypes *ikeda* and *chitose*, respectively, based on comparisons with reference sequences [GenBank: JQ781071, JQ781070, KC915202, KC915200]. Profile 3 represented a mix of profiles 1 and 2 (cf. [15,17]) and, consequently, was a mix of the profiles representing genotypes *ikeda* and *chitose* (Table 2). Therefore, the dominant genotype in all cows was *ikeda* (97.7%), followed by *chitose* (1.7%) (Table 2).

**Table 2:** **A summary of molecular test results and genotypes of *T. orientalis *** **detected in cows**

**Bleed 1**			**Bleed 2**		
**Molecular test result**	**Proportion of cows (%)**	**Genotype(s) detected (%; proportion)**	**Molecular test result**	**Proportion of cows (%)**	**Genotype(s) detected (%; proportion)**
Negative	361/662 (54.5%)		Negative	123/124 (99.2)	
			Positive	1/124 (0.8)	*Ikeda* (1.1; 1/92)
Positive	301/662 (45.5%)	*Ikeda* (97.7; 294/301)	Negative	5/96 (5.2)	
			Positive	91/96 (94.8)	*Ikeda* (97.8; 90/92); *Chitose* (1.1; 1/92); Mixed infection of *ikeda* and *chitose* (0; 0/92)
		*Chitose* (1.7; 5/301)			
		Mixed infection of *ikeda* and *chitose* (0.6; 2/301)			

‘Positive’ denotes that the blood DNA samples were fully characterised by PCR and sequencing and were found to be test-positive for *T. orientalis.*

To assess whether there was a change in the percentage of cows with *T. orientalis* infection in the period from November 2012 to March 2013, blood samples (*n* = 220) from the second sampling were tested molecularly and compared with molecular test results for the first sampling. Of 220 samples, 92 were test-positive for *T. orientalis* (Table 2), and genotypes *ikeda* and *chitose* were detected in 91 and 1 of the 92 samples, respectively; only one cow that was test-negative for *T. orientalis* in the first sampling was test-positive in the second. Similarly, five cows that were test-positive in the first sampling were test-negative in the second (Table 2). For one cow, genotype *ikeda* was identified in the first sampling and genotype *chitose* (but not *ikeda*) in the second. Conversely, for another cow, genotype *chitose* was identified in the first sampling and genotype *ikeda* (but not *chitose*) in the second. This “switch” in genotype was confirmed by PCR-based DNA sequencing of both *MPSP* and *SSU* genes (data not shown).

### Haematocrit values

An analysis of 603 blood samples (i.e., blood samples from all 603 cows with demographic data) from the first sampling showed that PCVs were significantly lower for group 1 (*P* < 0.001) and group 2 (*P* < 0.001) compared with group 4 cows. However, PCV for group 3 was not significantly lower (*P* = 0.087) compared with group 4 (Table 3).

**Table 3:** A comparison of packed cell volume (PCV) (in %) among different categories of dairy cows

**Variable**	**PCV**			
	**Group ( *n *)**	**Mean difference**^**a **^**(Standard error)**	**95% Confidence interval**^**a**^	***P*-value**^**a**^
Clinical and molecular test results	1 (16)	-14.36 (0.78)	-15.88, –12.83	<0.001
	2 (14)	-4.48 (0.83)	-6.11, –2.86	<0.001
	3 (254)	0.45 (0.26)	-0.06, 0.96	0.087
	4 (319)	Reference group		
Age (years)	≥ 6 (193)	1.77 (0.39)	1.02, 2.52	<0.001
	3-5 (309)	1.93 (0.35)	1.24, 2.62	<0.001
	≤ 2 (101)	Reference group		

Intercept for PCV was 24.9%.

^a^Mean difference was adjusted for three age categories (i.e., two years, three to five years, and six years and above).

Group 1, with cardinal clinical signs of oriental theileriosis & molecular test-positive for *T. orientalis*; group 2, with mild or suspected signs of theileriosis & test-positive for *T. orientalis*; group 3, with no clinical signs & test-positive for *T. orientalis*; and group 4, with no clinical signs & test-negative for *T. orientalis*.

*P*-values were obtained by comparing each category with the reference group.

### Production losses

Based on clinical signs and molecular test results (November 2012), group 1 comprised 16 diseased cows infected and affected by the genotype *ikeda*; group 2 consisted of 13 and one cows that were infected and mildly affected by genotypes *ikeda* and *chitose*, respectively; group 3 comprised 238 and four asymptomatic cows harbouring genotypes *ikeda* and *chitose*, respectively, and two other asymptomatic cows both harboured *ikeda & chitose*; group 4 comprised 309 asymptomatic cows with no evidence from molecular testing of *T. orientalis* infection*.* At 100 and 305 days of lactation, milk production data from cow groups 1–3 were compared (in a pairwise manner) with those from group 4. At 305 days, the numbers of cows in groups 1, 3 and 4 reduced slightly to 13, 221 and 289, respectively, since the rest of the cows had “dried-off” or were sold.

Analyses showed that, at 100 days of lactation, group 1 cows produced significantly less milk (288 l; *P* = 0.001) (Table 4), milk fat (16.8 kg; *P* < 0.001) (Table 5) and milk protein (12.6 kg; *P* < 0.001) than those in group 4 (Table 6). Group 2 cows also produced significantly less milk fat (13.6 kg; *P* = 0.002) (Table 5) and milk protein (8.6 kg; *P* = 0.005) than group 4 cows at this lactation point (Table 6).

**Table 4:** A comparison of milk volume (l) among different categories of dairy cows

**Variable**		**Milk volume at 100 days of lactation**				**Milk volume at 305 days of lactation**		
	**Group ( *n *)**^**a**^	**Mean difference**^**b **^**(Standard error)**	**95% Confidence interval**^**b**^	***P*-value**^**b**^	**Group ( *n *)**^**a**^	**Mean difference**^**b **^**(Standard error)**	**95% Confidence interval**^**b**^	***P*-value**^**b**^
Clinical and molecular test results	1 (16)	-288 (90.4)	-465.7, -111.2	0.001	1 (13)	-624 (216.9)	-1048.7, –198.6	0.004
	2 (14)	-153 (95.9)	-341.0, 35.0	0.11	2 (14)	-115 (208.6)	-523.7, 293.9	0.58
	3 (244)	11 (30.7)	-48.9, 71.4	0.72	3 (221)	82 (69.7)	-55.0, 218.1	0.24
	4 (309)	Reference group			4 (289)	Reference group		
Age (years)	≥ 6 (184)	595 (45.1)	506.4, 683.3	<0.001	≥ 6 (158)	1160 (101.3)	961.6, 1358.6	<0.001
	3-5 (302)	484 (41.6)	402.3, 565.4	<0.001	3-5 (283)	867 (91.3)	688.0, 1046.0	<0.001
	≤ 2 (97)	Reference group			≤ 2 (96)	Reference group		

Intercepts for milk volumes produced at 100 and 305 days of lactation were 1860 l and 4600 l, respectively.

^a^Numbers of cows differed, because some cows “dried off” or were sold.

^b^Mean difference was adjusted for three age categories (i.e., two years or less, three to five years, and six years or more).

Group 1, with cardinal clinical signs of oriental theileriosis & molecular test-positive for *T. orientalis*; group 2, with mild or suspected signs of theileriosis & test-positive for *T. orientalis*; group 3, with no clinical signs & test-positive for *T. orientalis*; and group 4, with no clinical signs & test-negative for *T. orientalis*.

*P*-values were obtained by comparing each category with the reference group.

**Table 5:** A comparison of milk fat (kg) among different categories of dairy cows

**Variable**		**Milk fat at 100 days of lactation**				**Milk fat at 305 days of lactation**		
	**Group ( *n *)**^**a**^	**Mean difference**^**b **^**(Standard error)**	**95% Confidence interval**^**b**^	***P*-value**^**b**^	**Group ( *n *)**^**a**^	**Mean difference**^**b **^**(Standard error)**	**95% Confidence interval**^**b**^	***P*-value**^**b**^
Clinical and molecular test results	1 (16)	-16.8 (4.2)	-25.0, –8.6	<0.001	1 (13)	-42.9 (10.4)	-63.2, –22.6	<0.001
	2 (14)	-13.6 (4.4)	-22.3, -4.9	0.002	2 (14)	-21.2 (10.0)	-40.7, –1.7	0.033
	3 (244)	1.0 (1.4)	-1.8, 3.7	0.50	3 (221)	2.2 (3.3)	-4.4, 8.7	0.51
	4 (309)	Reference group			4 (289)	Reference group		
Age (years)	≥ 6 (184)	23.0 (2.1)	18.9, 27.1	<0.001	≥ 6 (158)	49.3 (4.8)	39.8, 58.8	<0.001
	3-5 (302)	17.8 (1.9)	14.0, 21.6	<0.001	3-5 (283)	37.6 (4.4)	29.0, 46.1	<0.001
	≤ 2 (97)	Reference group			≤ 2 (96)	Reference group		

Intercepts for milk fat produced at 100 and 305 days of lactation were 79.0 kg and 213.3 kg, respectively.

^a^Numbers of cows differed because some cows “dried off” or were sold.

^b^Mean difference was adjusted for three age categories (i.e., two years or less, three to five years, and six years or more).

Group 1, with cardinal clinical signs of oriental theileriosis & molecular test-positive for *T. orientalis*; group 2, with mild or suspected signs of theileriosis & test-positive for *T. orientalis*; group 3, with no clinical signs & test-positive for *T. orientalis*; and group 4, with no clinical signs & test-negative for *T. orientalis*.

*P*-values were obtained by comparing each category with the reference group.

**Table 6:** A comparison of milk protein (kg) among different categories of dairy cows

**Variable**		**Milk protein at 100 days of lactation**				**Milk protein at 305 days of lactation**		
	**Group ( *n *)**^**a**^	**Mean difference**^**b **^**(Standard error)**	**95% Confidence interval**^**b**^	***P*-value**^**b**^	**Group ( *n *)**^**a**^	**Mean difference**^**b **^**(Standard error)**	**95% Confidence interval**^**b**^	***P*-value**^**b**^
Clinical and molecular test results	1 (16)	-12.6 (2.9)	-18.2, -7.0	<0.001	1 (15)	-26.0 (7.0)	-39.7, -12.3	<0.001
	2 (14)	-8.6 (3.0)	-14.5, -2.7	0.005	2 (14)	-11.1 (6.7)	-24.3, 2.1	0.099
	3 (244)	0.30 (1.0)	-1.6, 2.2	0.76	3 (219)	2.9 (2.2)	-1.5, 7.3	0.20
	4 (309)	Reference group			4 (289)	Reference group		
Age (years)	≥ 6 (184)	20.7 (1.4)	18.0, 23.5	<0.001	≥ 6 (158)	39.6 (3.3)	33.2, 46.0	<0.001
	3-5 (302)	17.2 (1.3)	14.6, 19.8	<0.001	3-5 (283)	31.6 (2.9)	25.8, 37.4	<0.001
	≤ 2 (97)	Reference group			≤ 2 (96)	Reference group		

Intercepts for milk protein produced at 100 and 305 days of lactation were 61.5 kg and 160.0 kg, respectively.

^a^Numbers of cows differed, because some cows “dried off” or were sold.

^b^Mean difference was adjusted for three age categories (i.e., two years or less, three to five years, and six years or more).

Group 1, with cardinal clinical signs of oriental theileriosis & molecular test-positive for *T. orientalis*; group 2, with mild or suspected signs of theileriosis & test-positive for *T. orientalis*; group 3, with no clinical signs & test-positive for *T. orientalis*; and group 4, with no clinical signs & test-negative for *T. orientalis*.

*P*-values were obtained by comparing each category with the reference group.

Analyses also showed that, at 305 days of lactation, group 1 cows produced significantly less milk (624 l; *P* = 0.004) (Table 4), milk fat (42.9 kg; *P* < 0.001) (Table 5) and milk protein (26.0 kg; *P* < 0.001) compared with group 4 cows (Table 6). Group 2 cows also produced significantly less milk fat (21.2 kg; *P* = 0.033) (Table 5) at this lactation point. However, no significant difference in milk production was found between group 3 and group 4 cows at both 100 and 305 days of lactation (Tables 4, 5 and 6)*.* In contrast to the significant decrease in milk production observed for groups 1 and 2, no statistically significant difference in reproductive performance was found upon pairwise comparisons of groups 1–3 with group 4 cows (Additional file 1: Table S1 and Additional file 2: Table S2).

## Discussion

The present findings showed that, while reproduction was not significantly affected, dairy cows suffering from clinically severe oriental theileriosis (group 1) produced 624 l less milk per head at 305 days of lactation, leading to an estimated annual economic loss of AUD 202 (USD 179) per head. The loss caused by the death of a single cow was AUD 1,800. Therefore, on the farm studied here, the total economic loss associated with 16 severely affected cows and one dead cow was estimated at AUD 5,035 in the first year. Although not statistically significant, the reduction in milk production in the 14 cows inferred to be mildly affected by *T. orientalis* infection was 115 l per head at 305 days of lactation, which suggests that a further AUD 522 may have been lost. Although the mortality rate due to oriental theileriosis was low in the present study, rates as high as 11% have been recorded in the state of Victoria, Australia (J. M., personal communication; December 2013). This loss is proposed to be much less in subsequent years due to an apparent “stabilisation” of the infection or immunity of the herd. From the current study, it is apparent that molecular testing is critical to assessing the status of oriental theileriosis in herds.

Previously, no information was available on the production losses associated with clinical form of oriental theileriosis for any part of the world, including Australia, in which numerous outbreaks have occurred in the past two years. Nonetheless, production losses linked to other species of *Theileria* in bovines, such as *T. parva*, have been recorded in Kenya [24,25] and in Tanzania [26-28]. In addition, production losses and mortality rates associated with theileriosis caused by *T. annulata* have been reported from Turkey [29].

In Australia in 1997, economic losses caused by ticks and tick-borne diseases in cattle were reported to cost USD 7.8 per head [30], which is likely to be considerably higher today*.* In 1999, tick fever caused by *Babesia bovis*, *Babesia bigemina* and/or *Anaplasma marginale* cost the beef industry in northern Australia AUD 27 million [31]. The annual cost per head caused by oriental theileriosis is likely to be significant, and seems to be similar to the annual cost of AUD 227 per head due to *T. parva* in Tanzania [28]. However, there is a limitation in comparing studies, because the present study was based on both clinical diagnosis and molecular identification of the parasite, while the study by Kivaria *et al.*[28] relied on clinical signs and microscopy for the diagnosis of theileriosis. In addition, the economic losses estimated by these authors [28] were calculated by adding the costs of milk loss, weight loss, acaricidal treatment and immunisation [28], but the present study considered only the loss in milk production. However, if the cost of tick control were included here, the total loss per head due to oriental theileriosis would likely to be considerably higher.

In the present study, we found that the clinical outbreak was linked mainly to a high (97.7%) and low (1.7%) prevalence of the virulent genotypes, *ikeda* and *chitose*, of *T. orientalis*, respectively. Sequences representing these two genotypes were the same as those [GenBank: JQ781071, JQ781070, KC915200, KC915202] characterised previously for *T. orientalis* from cattle in Victoria [15,17]. Although the genotype *buffeli* of *T. orientalis* is suspected to be endemic to some parts of Gippsland region (C. M. Bell, unpublished data), we did not detect this genotype herein using the present molecular approach. The high prevalence of genotype *ikeda* in cows on the present ‘closed’ dairy farm may have occurred as a consequence of cattle movement to nearby properties (during the dry period) from other regions in Victoria, where this genotype is prevalent. In addition, this might relate to a dominance of the virulent genotype populations over ‘benign’ types of *T. orientalis*[16].

Genotypes found in the bloods of individual cows did not alter from the first to second sampling, except for two cows. However, changes in a dominant parasite population have been reported to occur during the transmission from calves to ticks and from infected ticks to calves [32]. Changes in *ikeda* and *chitose* populations might also relate to host immune pressure on the parasite during persistent infection over several months [32]. Although this evidence might explain a possible genotypic switch, further, detailed investigations are required to establish whether genotypic alterations do actually occur within a period as short as three months (as in the present study).

Here, the number of cows with *T. orientalis* infection in the herd was relatively stable; five test-positive cows became test-negative, and one test-negative cow became test-positive within the study period of three-months. Previously, a study by Shimizu *et al.*[33] also showed that new infections (87.5% of 8) by *T. orientalis* occurred within a period of two months. However, there are limitations in comparing the present study with that of Shimizu *et al.*[33], as the latter authors used ELISA for the indirect detection of infection (through serum antibody), whereas we used a more sensitive method (PCR-based mutation scanning and selective sequencing) for the direct detection of parasite DNA. Similar to the findings of the present study, Ota *et al.*[8] reported that the prevalence of *T. orientalis* infection increased from 2.2% of 89 to up to 48.4% of 91 by three weeks, and up to 64.8% of 91 cattle (various breeds) by two months, based on testing by PCR and sequencing of two loci (i.e., *MPSP* and *p23*). These authors [8] suggested that the possible reason for new infections could be the seasonal activity of the infected tick vector. In the present study, new *T. orientalis* infections could have occurred as a result of tick transmission from other infected cows in the herd; however, the role of ticks in the transmission of oriental theileriosis in Victoria remains enigmatic and requires detailed study.

## Conclusions

Although economic losses associated with oriental theileriosis appear to have been ignored in the past, the present findings clearly show that clinical oriental theileriosis can cause significant milk production losses in dairy cattle. Future investigations should focus on assessing the economic impact of this disease on a much larger scale in eastern Australia. In the meantime, efforts should focus on improving the management and control of oriental theileriosis to subvert the impact of this disease on the dairy industry in Australia.

## Abbreviations

CMSD: Calving-to-mating start date; CMSDsq: Calving-to-MSD squared; CR1: First service conception rate; CS1: Calving-to-service interval; CS1sq: CS1 squared; ICR6w: In-Calf by 6 weeks; ICR14w: In-Calf by 14 weeks; MPSP: Major piroplasm surface protein; PCV: Packed cell volume; NIC21w: Not In-Calf at 21 weeks; SSU rRNA: Small subunit of nuclear ribosomal RNA; SR21d: Submission rate by 21 days.

## Competing interests

The authors declare that they have no competing interests.

## Authors’ contributions

PP carried out the PCR-based analysis, data analyses, interpretation of data and also drafted the manuscript, with guidance from co-authors. JM and GD undertook clinical examinations of cows and participated in the study design. DSB and JM provided milk production data. JM and GD collected blood samples and determined PCVs. SMF, GAA and PP performed the statistical analyses. AJ, RBG and JM conceived the project, participated in the study design, interpreted the data and provided critical inputs on the draft manuscript. All authors read and approved the final manuscript.

## Supplementary Material

Additional file 1: Table S1 A comparison of reproduction indices among different categories of dairy cows. Click here for file

Additional file 2: Table S2 A comparison of reproduction indices among different categories of dairy cows. Click here for file
