# Supplementary material for: Oriental theileriosis in dairy cows causes a significant milk production loss
Source: Parasit Vectors. 2014 Feb 19;7:73. doi: 10.1186/1756-3305-7-73 (PMC3937217; doi:10.1186/1756-3305-7-73)
Supplement: Additional file 1: Table S1 — A comparison of reproduction indices among different categories of dairy cows. [file 1756-3305-7-73-S1.doc]

Supplementary tables

Table S1 A comparison of reproduction indices among different categories of dairy cows

| *Variable First service conception*  *rate (CR1)* | | | | | *In-calf by 6 weeks*  *(ICR6w)* | | | *In-calf by 14 weeks (ICR14w)* | | | | |
| --- | --- | --- | --- | --- | --- | --- | --- | --- | --- | --- | --- | --- |
|  | Group (*n*)^a^ | Odds ratio^b^ | 95% CI^b^ | *P*-value^b^ | | Odds ratio^c^ | 95% Confidence Interval^c^ | | *P*-value^c^ | Odds ratio^c^ | 95% Confidence Interval^c^ | *P*-value^c^ |
| Clinical and molecular test results |  |  |  |  | |  |  | |  |  |  |  |
|  | 1 (16) | 0.88 | 0.31, 2.44 | 0.80 | | 0.87 | 0.30, 2.53 | | 0.80 | 0.58 | 0.15, 2.22 | 0.43 |
|  | 2 (14) | 0.76 | 0.26, 2.27 | 0.62 | | 0.52 | 0.17, 1.57 | | 0.24 | 1.95 | 0.24, 15.88 | 0.53 |
|  | 3 (254) | 1.27 | 0.90, 1.80 | 0.17 | | 1.08 | 0.74, 1.57 | | 0.70 | 0.67 | 0.40, 1.13 | 0.13 |
|  | 4 (319) | Reference group | | | | Reference group | | | | Reference group | | |

^a^Different numbers in the groups relate to CR1.

^b^ Odds ratio was adjusted for three age categories (i.e., two years or less, three to five years, and six years and above), calving to service interval (CS1) and calving to service interval squared (CS1sq).

^c^ Odds ratio was adjusted for three age categories, calving-to-mating start date (CMSD) and calving-to-mating start date squared (CMSDsq).

Group 1, with cardinal clinical signs of oriental theileriosis & molecular test-positive for *T. orientalis*; group 2, with mild or suspected signs of theileriosis & test-positive for *T. orientalis*; group 3, with no clinical signs & test-positive for *T. orientalis*; and group 4, with no clinical signs & test-negative for *T. orientalis*.

*P*-values were obtained by comparing each category with the reference group.
