# Supplementary material for: Oriental theileriosis in dairy cows causes a significant milk production loss
Source: Parasit Vectors. 2014 Feb 19;7:73. doi: 10.1186/1756-3305-7-73 (PMC3937217; doi:10.1186/1756-3305-7-73)
Supplement: Additional file 2: Table S2 — A comparison of reproduction indices among different categories of dairy cows. [file 1756-3305-7-73-S2.doc]

Table S2 A comparison of reproduction indices among different categories of dairy cows

| *Variable* | *Not in calf at 21 weeks (NIC21w)* | | | *Submission rate by 21 days (SR21d)* | | | |
| --- | --- | --- | --- | --- | --- | --- | --- |
|  | Group (*n*) | Odds ratio^a^ | 95% Confidence Interval^a^ | *P*-value^a^ | Odds ratio^a^ | 95% Confidence Interval^a^ | *P*-value^a^ |
| Clinical and molecular test results | 1 (16) | 2.26 | 0.58, 8.79 | 0.24 | 1.07 | 0.23, 4.98 | 0.94 |
|  | 2 (14) | 0.69 | 0.08, 5.69 | 0.73 | 0.58 | 0.15, 2.25 | 0.43 |
|  | 3 (254) | 1.43 | 0.80, 2.58 | 0.23 | 1.02 | 0.61, 1.73 | 0.94 |
|  | 4 (319) | Reference group | | | Reference group | | |

^a^ Odds ratio was adjusted according to age category (i.e., ≤ 2 years, 3-5 years, and ≥ 6 six years), calving to mating start date (CMSD) and calving to mating start date squared (CMSDsq).

Group 1, with cardinal clinical signs of oriental theileriosis & molecular test-positive for *T. orientalis*; group 2, with mild or suspected signs of theileriosis & test-positive for *T. orientalis*; group 3, with no clinical signs & test-positive for *T. orientalis*; and group 4, with no clinical signs & test-negative for *T. orientalis*.

*P*-values were obtained by comparing each category with the reference group.
